# Quality of Life Assessment Using EuroQOL EQ-5D Questionnaire in Patients with Deep Infiltrating Endometriosis: The Relation with Symptoms and Locations

**DOI:** 10.1155/2013/452134

**Published:** 2013-07-24

**Authors:** C. Touboul, P. Amate, M. Ballester, M. Bazot, A. Fauconnier, E. Daraï

**Affiliations:** ^1^Service de Gynécologie-Obstétrique, GRC-UPMC 6: Centre Expert en Endometriose (C3E), Hôpital Tenon, Assistance Publique des Hôpitaux de Paris, Université Pierre et Marie Curie Paris VI, 4 rue de la Chine, 75020 Paris, France; ^2^Service de Gynécologie-Obstétrique, Hôpital Tenon, 4 rue de la Chine, 75020 Paris, France; ^3^Service de Radiologie, Hôpital Tenon, Assistance Publique des Hôpitaux de Paris, Université Pierre et Marie Curie Paris VI, 4 rue de la Chine, 75020 Paris, France; ^4^Service de Gynécologie-Obstétrique, Centre Hospitalier Interrégional de Poissy, Université Paris XIV Saint Quentin en Yvelynes, 10 Rue du Champ Gaillard, 78300 Poissy, France

## Abstract

The objective of this study was to evaluate the quality of life (QOL) of patients with deep infiltrating endometriosis (DIE) using EuroQOL (EQ-5D) and its correlation with symptoms and locations of endometriotic lesions. One hundred and fifty-nine patients referred for DIE from January 2011 to April 2011 were prospectively invited to complete questionnaires evaluating symptoms associated with endometriosis as well as the EQ-5D questionnaire and health state. Patients also had locations of DIE evaluated by clinical examination and magnetic resonance imaging (MRI). All 159 patients completed the questionnaires. Item response rate was 91.1%. The most intense symptoms were dysmenorrhea (7.1/10), painful defecation (6.3/10), and fatigue (6.0/10). Mean (SD) scores were 77 (14) for the EQ-5D questionnaire and 63.4 (21) for the health state. A relation was observed between the EQ-5D questionnaire and the presence of dysmenorrhea, dyspareunia, cyclic pelvic pain, painful defecation, and diarrhea or constipation. Vaginal and rectal infiltrations were significantly associated with altered EQ-5D and health state scores. The EQ-5D questionnaire is easy to complete and well related to symptoms of DIE. Rectal and vaginal infiltrations were found to be determinant factors of altered QOL by the EQ-5D questionnaire and health state.

## 1. Introduction

Endometriosis is a benign but potentially debilitating gynecologic disorder affecting 10% to 15% of patients during their reproductive life [1]. It is a well-known cause of chronic pelvic pain, dysmenorrhea, and dyspareunia affecting quality of life (QOL) [2–4]. There are three types of endometriosis defined by the presence of endometrial glands and stroma outside the uterus: the peritoneal, the ovarian, or the deep infiltrating endometriosis (DIE); this latter type is characterized by involvement of organs and pelvic anatomic structures by endometriotic lesions. 

Specific multidimensional questionnaires to evaluate QOL in patients with endometriosis have been developed—by Bodner et al. in 1997 [2], Colwell et al. in 1998 [3], and Jones et al. in 2001 [4]—but have only been validated in English. Nonspecific multidimensional QOL questionnaires such as the short form health survey SF-36 have also been developed and validated in various languages allowing comparison between countries [5, 6]. Despite the absence of specificity for the population of patients with endometriosis, the SF-36 questionnaire has been demonstrated to be a useful tool to select patients with colorectal endometriosis for surgery and to evaluate long-term functional results [7–9]. However, both the SF-36 and the Endometriosis Health Profile-30 (EHP-30) questionnaire are time consuming for the responders and difficult to interpret due to the high number of items. The EuroQOL (EQ-5D) is a five-question QOL questionnaire, the final version of which was developed in 1991 [10]. It has been used to evaluate QOL in patients with chronic pelvic pain and endometriosis [11–13]. Although it was translated into various languages (including French) between 1992 and 1995, thus allowing cross-national comparison of QOL, it has not been validated in patients with DIE.

The aim of this prospective study was, therefore, to evaluate the QOL of patients with DIE using EQ-5D and its correlation with symptoms and locations of endometriotic lesions.

## 2. Material and Methods

### 2.1. Population

From 20 January to 24 April, 2011, all patients referred to our tertiary university center for suspected DIE were prospectively invited to fill out the French version of the EQ-5D questionnaire [10].

All patients underwent a clinical examination followed by magnetic resonance imaging (MRI) with a referent radiologist Marc Bazot (M.B). Diagnostic criteria for DIE were those previously published [14–18]. Patients with prior surgery for DIE were excluded. All data were collected before any potential surgical treatment of DIE. The study was approved by the Ethics Committee of the Collège National des Gynécologues et Obstétriciens Français (no. CEROG-2011-GYN-03-03-R1). 

### 2.2. Evaluation of Symptoms and QOL

A previously published and validated questionnaire was used to evaluate the patients' symptoms [18]. This questionnaire takes into account the presence of gynecological symptoms (dysmenorrhea, dyspareunia, and cyclic pelvic pain), digestive symptoms (diarrhea, constipation, rectal bleeding, abdominal cramping, and painful defecation), urinary symptoms (urgentury, urinary incontinence, hematuria, and incomplete emptying), and general symptoms (back pain and fatigue). In addition to the presence of the symptoms, intensity was evaluated using a visual analogic scale (0 for no pain and 10 for unbearable pain). 

The French version of the five-question (item) EQ-5D questionnaire and the global health state were used to evaluate QOL. The five items include mobility, self-care, daily activities, pain, and emotional well-being (anxiety or depression). Each item is rated on a three-point scale (never = 0, sometimes = 1, and always = 2). The EQ-5D score was calculated by adding the scores of the five questions giving a range from 0 to 10. Scores were then transformed onto a scale of 0 (indicating best possible status) to 100 (indicating worst possible status). The EQ-5D also included a global health state measurement on a visual analytic scale (VAS) ranging from 0 to 100. 

### 2.3. Statistical Analysis

Univariate comparisons of QOL data were conducted with the use of a 2-sample *t*-test or Wilcoxon test for continuous data and a Chi^2^ test or Fisher's exact test for categorical data. Pearson test was used to study the correlation between two continuous variables. Univariate analysis was also used to identify factors associated with altered QOL (defined as worst quartile). All reported *P* values were 2 sided. A *P* value of less than 0.05 denotes a significant difference. 

## 3. Results

### 3.1. Epidemiological Characteristics of the Study Population

Two hundred and twenty-three patients consulted for clinical suspicion of DIE during the study period. Sixty-four patients were excluded because they had already undergone a resection of DIE leaving a study population of 159 patients all of whom accepted to fill out the EQ-5D questionnaire. The questionnaire response rate was 100% and item response rate 91.1% (145 out of 159 patients completed all of the core questionnaire items). The missing response rates for each item in the core section ranged from 1% to 2%. The EQ-5D health status response rate was 83% (27 missing data).

The mean (SD) age of the patients was 34.8 years (6.6). The mean (SD) body mass index (BMI) was 22.8 kg/m^2^ (4.4). The mean (SD) gravidity was 0.9 pregnancies (1.6) and parity 0.6 children (0.9). Fifty-eight patients (36.5%) were infertile with a mean duration of 4.1 (3.3) years. One hundred of the 159 patients (63%) had a previous laparoscopic diagnosis of endometriosis with histologic confirmation. The remaining 59 patients had a diagnosis of DIE based on MRI criteria.

The most frequent symptoms reported by the patients were dysmenorrhea (89%), diarrhea or constipation (69%), dyspareunia (66%), cyclic pelvic pain (65%), painful defecation (58%), rectal bleeding (16%), and urinary symptoms (14%) (Table 1). The most intense symptoms were dysmenorrhea (7.1/10), painful defecation (6.3/10), and fatigue (6.0/10) (Table 1). 

### 3.2. Locations of DIE Lesions

Distribution of the DIE lesions on clinical examination and MRI is given in Table 2.

On clinical examination, the most frequent locations of DIE were the torus uterinum (50%) and the uterosacral ligaments (42%). Twenty-seven percent of the patients had vaginal endometriosis and 28% colorectal endometriosis. Parametrial involvement was diagnosed by rectal digital examination in 17% of cases. There was no suggestion of adenomyosis or bladder endometriosis. 

On MRI, the most frequent locations of DIE were also the torus uterinum (69%) and the uterosacral ligaments (69%) followed by the colorectum (48%). Parametrial involvement was found in 12% of the cases. In addition to DIE lesions, endometriomas were found in 36% of the patients.

### 3.3. Relation between Symptoms and Quality of Life

The mean (SD) score of the EQ-5D questionnaire was 77 (14). A relation was found between the EQ-5D score and the presence of dysmenorrhea (*P* = 0.01), dyspareunia (*P* < 0.001), cyclic pelvic pain (*P* < 0.001), painful defecation (*P* < 0.001), and diarrhea or constipation (*P* < 0.001). No relation was found between the EQ-5D score and the presence of rectal bleeding or urinary symptoms (Table 3).

The mean (SD) score of the EQ-5D questionnaire in patients with or without infertility was 22.8 and 23.8, respectively. The EQ-5D score was not significantly altered in patients with infertility or correlated with patient's age.

The mean (SD) health state was 63.4 (21.0), and a relation was found between the EQ-5D health state and the presence of dysmenorrhea (*P* = 0.03), dyspareunia (*P* < 0.01), cyclic pelvic pain (*P* < 0.001), painful defecation (*P* = 0.01), and diarrhea or constipation (*P* < 0.01). No relation was found between the EQ-5D health state and the presence of rectal bleeding or urinary symptoms (Table 3). 

The mean (SD) value of the EQ-5D health state in patients with or without infertility was 56.3 and 60.6, respectively. It was not significantly altered in patients with infertility or correlated with patient's age.

Pearson's test revealed a relation between the EQ-5D score and the intensity of dysmenorrhea (*P* < 0.001, correlation coefficient (cor) = 0.33 (95% CI = 0.18–0.47)), dyspareunia (*P* < 0.001, cor = 0.45 (95% CI = 0.31–0.57)), abdominal cramping (*P* < 0.001, cor = 0.43 (95% CI = 0.30–0.56)), painful defecation (*P* = 0.02, cor = 0.39 (95% CI = 0.24–0.52)), diarrhea or constipation (*P* < 0.01, cor = 0.34 (95% CI = 0.19–0.48)), dysuria (*P* < 0.001, cor = 0.31 (95% CI = 0.15–0.45)), urinary urgency (*P* < 0.01, cor = 0.23 (95% CI = 0.07–0.38)), and fatigue (*P* < 0.001, cor = 0.42 (95% CI = 0.28–0.55)). No relation was found between the EQ-5D score and the intensity of cyclic pelvic pain or rectal bleeding. 

An association between a low QOL and dysmenorrhea, dyspareunia, chronic pelvic pain, diarrhea, constipation, and painful defecation was found using the worst quartile of the EQ-5D score. 

Pearson's test revealed a relation between the EQ-5D health state and the intensity of dysmenorrhea (*P* < 0.001, cor = 0.34 (95% CI = 0.17–0.49)), dyspareunia (*P* < 0.001, cor = 0.30 (95% CI = 0.12–0.45)), abdominal cramping (*P* < 0.001, cor = 0.38 (95% CI = 0.22–0.52)), painful defecation (*P* = 0.01, cor = 0.29 (95% CI = 0.12–0.45)), diarrhea or constipation (*P* = 0.01, cor = 0.21 (95% CI = 0.04–0.38)), dysuria (*P* < 0.01, cor = 0.23 (95% CI = 0.05–0.38)), and fatigue (*P* < 0.001, cor = 0.49 (95% CI = 0.35–0.62)). No relation was found between the EQ-5D health state and the intensity of cyclic pelvic pain, rectal bleeding, or urinary urgency. 

Using the worst quartile of the EQ-5D health state, a decrease in QOL was associated with dysmenorrhea, dyspareunia, chronic pelvic pain, constipation, abdominal cramping, and painful defecation.

A relation was found between a decrease in QOL using the worst quartile of EQ-5D score and rectal or vaginal involvement. Similarly, a relation was found between a decrease in QOL using the EQ-5D health state and rectal or vaginal involvement.

## 4. Discussion

The present study demonstrates the relevance of the EQ-5D questionnaire in evaluating QOL in patients with DIE. A relation was found between the presence and the intensity of symptoms reported by patients with DIE and the alteration in QOL. Moreover, vaginal and rectal endometrioses were determinant factors of altered QOL.

Although the evaluation of QOL in patients with DIE is a major issue, a recent review by Meuleman et al. including 49 series of patients undergoing surgery for DIE with colorectal involvement found that only 10% of them evaluated QOL [19]. This low rate could be partly related to the absence of consensus about the most reliable and simple QOL questionnaire to use. The strengths of EQ-5D questionnaire lie in its simplicity (easy to complete by respondents) and its availability in several languages [10]. In contrast, despite the high reliability of EHP-30 questionnaire to evaluate QOL in English patients [20, 21], only a few translations are available in other languages so that comparison between countries is not possible [22]. Moreover, similar to the SF-36 questionnaire, the EHP-30 questionnaire is time consuming and difficult to interpret due to the high number of items which explains its limited use in trials and routine practice. 

The current study is the first validation of EQ-5D questionnaire in a series of patients with DIE. The high proportion of patients with severe DIE (nearly half of the patients with colorectal endometriosis and one-third with vaginal infiltration at MRI) can be explained by the recruitment of our department including an expert center in endometriosis. Moreover, at clinical examination, 17% of the patients had parametrial involvement. Hence, these data explained the high incidence of urinary and digestive symptoms observed, respectively, in 30% and 50% of our population. Data completeness was very high with a low missing item response rate supporting the reliability of EQ-5D questionnaire. These results of completeness are in agreement with those of Khong et al. [23] confirming that the EQ-5D questionnaire is suitable as a self-report health status instrument for patients with DIE. Moreover, our results are in agreement with those of Abbott et al. [12] which show that the EQ-5D questionnaire is as satisfactory as the SF-12 questionnaire in evaluating QOL in patients with endometriosis. Finally, in a randomized trial evaluating laparoscopic uterosacral nerve ablation to alleviate chronic pelvic pain, Daniels et al. [13] confirmed that the EQ-5D questionnaire was a useful tool to evaluate QOL. 

The current study has demonstrated that patients with DIE had a significantly altered QOL with a mean EQ-5D score and EQ-5D health state of 77 and 63.4, respectively. These values correspond to those reported by Fourquet et al. [24] as reflecting the high impact of endometriosis. In addition, our results have demonstrated the relation between the EQ-5D score and EQ-5D health state and the presence of dysmenorrhea, dyspareunia, chronic pelvic pain, diarrhea, constipation, and painful defecation. These results are in agreement with those of previous studies showing a relation between the symptoms of patients with colorectal endometriosis and a decrease in QOL [9, 18, 25]. Souza et al. [26] showed that higher pain scores were correlated to lower QOL but that having endometriosis in addition to chronic pelvic pain did not have an additional impact. However, in contrast to our population, all the patients in their series presented minimal/mild endometriosis, and no data were available about the presence of DIE lesions. Nevertheless, our data are in keeping with those of Nnoaham et al. [27] who observed that physical QOL was significantly reduced in patients affected by endometriosis compared with those with similar symptoms and no endometriosis. Moreover, endometriosis had a high impact on work productivity which increased over time after surgical diagnosis [27]. This is also in accordance with previous studies [24, 28] reporting a high impact of endometriosis on work, with loss in work time (absenteeism), impaired work (presenteeism), loss in efficiency levels (work productivity loss), and perturbed daily activities (activity impairment).

In addition to symptoms, previous studies have highlighted several factors potentially impacting QOL. Indeed, a potential negative impact of infertility on QOL has been reported [29–32]. Using the EHP-5 questionnaire, Goshtasebi et al. [33] found that the coexistence of infertility with symptoms of endometriosis was associated with a significant decrease in QOL compared to patients with endometriosis without infertility. These results are in contrast to our data, which show no relation between infertility or patient age and a decrease in the EQ-5D score and EQ-5D health state. This discrepancy can be explained by confounding factors. Khong et al. [23] found that altered QOL was more pronounced in young patients, with self-image deteriorating as the disease progresses with time. Moreover, these authors underlined that young patients were more concerned about fertility and worried that endometriosis may have caused irreversible damage on their reproductive organs. This also explains their observation that patients with children had better QOL scores than those who did not. However, none of these authors evaluated QOL in relation to the presence of DIE which represents the most severe form of endometriosis. In addition, our results suggest that some DIE lesions, such as rectal and vaginal endometriosis, are associated with a decrease in QOL. These results are in line with those of Fauconnier et al. who reported higher levels of pain when the endometrioses involved the bowel and a higher frequency of painful defecation during menstruation when DIE involved the vagina [34]. Chopin et al. also reported increased dysmenorrhea in the case of rectal infiltration [35]. All these considerations emphasize the importance of taking into account symptoms reported by patients, especially adolescents, to diagnose endometriosis as early as possible [36] and to provide information and psychosocial counseling which contributes to an improved QOL.

Some limitations of the present study have to be underlined. First, we focused on a subgroup of patients with DIE and cannot, therefore, draw conclusions about the relevance of the EQ-5D questionnaire in patients with minimal/mild or moderate endometriosis. Second, not all the patients included in the present study underwent histology to confirm the presence of endometriosis. However, as previously mentioned, our population was exclusively composed of patients with DIE, and in this specific setting previous studies on MRI have demonstrated its high accuracy in detecting endometriosis compared to clinical examination [15, 37]. Finally, we cannot exclude a potential bias linked to the specific recruitment of our department with a high rate of patients with colorectal endometriosis or extensive lesions explaining the high EQ-5D score and health status reported in the current study. 

## 5. Conclusion

The present prospective study supports the relevance of the EQ-5D questionnaire in evaluating QOL in patients with DIE. Further studies are required to demonstrate the relevance of this questionnaire in evaluating the impact of therapies especially after surgery and to identify good candidates for surgery as previously demonstrated using the SF-36 questionnaire.

## Figures and Tables

**Table 1:** Incidence and intensity of the symptoms reported by the 159 women with deep infiltrating endometriosis.

Symptoms	Women with symptoms (%)	Intensity of symptoms on VAS mean (SD∗)
Gynecological		
Dysmenorrhea	142 (89)	7.1 (2.3)
Dyspareunia	105 (66)	5.0 (2.6)
Gastrointestinal		
Diarrhea	67 (42)	5.2 (2.4)
Constipation	89 (56)	5.5 (2.6)
Rectal bleeding	26 (16)	3.6 (2.6)
Abdominal cramping	107 (67)	5.8 (2.6)
Painful defecation	92 (58)	6.3 (2.4)
Urinary		
Dysuria	23 (14)	4.5 (2.6)
Urgencies	52 (33)	5.9 (2.6)
Spontaneous urinary incontinence	21 (13)	4.5 (2.8)
Stress urinary incontinence	32 (20)	3.8 (2.5)
Incomplete emptying	62 (39)	4.6 (2.7)
General		
Fatigue	114 (72)	6.0 (2.4)
Back pain	64 (40)	5.5 (2.6)

^*^Standard deviation.

**Table 2:** Locations of deep infiltrating endometriosis on clinical examination and on MRI∗ of the 159 women.

	Clinical examination Number of women (%)	MRI∗ Number of women (%)
Torus	80 (50%)	110 (69%)
Uterosacral ligament	67 (42%)	109 (69%)
Adenomyosis	—	38 (24%)
Parametrium	27 (17%)	19 (12%)
Rectum	44 (28%)	79 (48%)
Sigmoid	—	10 (6%)
Caecum or ileon	—	4 (3%)
Vagina	43 (27%)	41 (26%)
Bladder	—	3 (2%)

^*^Magnetic resonance imaging.

**Table 3:** Correlation between the symptoms reported by the 159 women with deep infiltrating endometriosis and the EQ-5D score and EQ-5D Health State results in univariable analysis.

Symptom	EQ-5D score		EQ-5D health state	
	Value	*P* value	Value	*P* value
Dysmenorrhea				
Yes	23.9	0.01	61.2	0.03
No	11.5		76.6	
Dyspareunia				
Yes	26.7	<0.001	57.7	<0.01
No	17.1		68.2	
Cyclic pelvic pain				
Yes	26.7	<0.001	58.4	<0.001
No	15.1		71.0	
Painful defecation				
Yes	26.4	<0.001	59.1	0.01
No	17.8		67.7	
Gastrointestinal disorders				
Yes	25.8	<0.001	59.5	<0.01
No	15.9		69.9	
Rectal bleeding				
Yes	26.0	NS	67.7	NS
No	22.4		61.5	
Urinary disorders				
Yes	27.3	NS	60.6	NS
No	21.9		62.9	
